# Stable Isotope Dilution Analysis (SIDA) to Determine Metabolites of Furan and 2-Methylfuran in Human Urine Samples: A Pilot Study

**DOI:** 10.3390/metabo13091011

**Published:** 2023-09-14

**Authors:** Jonathan Isaak Kremer, Dorothea Karlstetter, Verena Kirsch, Daniel Bohlen, Carina Klier, Jan Rotermund, Hannah Thomas, Lukas Lang, Hanna Becker, Tamara Bakuradze, Simone Stegmüller, Elke Richling

**Affiliations:** Department of Chemistry, Division of Food Chemistry and Toxicology, RPTU Kaiserslautern-Landau, Erwin-Schrödinger-Str. 52, D-67663 Kaiserslautern, Germany; kremer@chemie.uni-kl.de (J.I.K.); karlstetter@chemie.uni-kl.de (D.K.); verenakirsch@gmx.de (V.K.); bohlen@chemie.uni-kl.de (D.B.); klier@rhrk.uni-kl.de (C.K.); rotermund@rhrk.uni-kl.de (J.R.); thomas@rhrk.uni-kl.de (H.T.); langl@rhrk.uni-kl.de (L.L.); hanna.becker@chem.rptu.de (H.B.); tamara.bakuradze@chem.rptu.de (T.B.); simone.stegmueller@chem.rptu.de (S.S.)

**Keywords:** furan, 2-methylfuran, toxicokinetic, *cis*-2-buten-1,4-dial, 3-acetyl acrolein, coffee, metabolism, SIDA

## Abstract

Furan and 2-methylfuran (2-MF) are food contaminants that are classified as potentially carcinogenic to humans. The main source of exposure for adults via food is coffee consumption. Furan and 2-MF are volatile, which complicates exposure assessment because their content measured in food prior to consumption does not afford a reliable dosimetry. Therefore, other ways of exposure assessment need to be developed, preferably by monitoring exposure biomarkers, e.g., selected metabolites excreted in urine. In this study, *cis*-2-buten-1,4-dial (BDA)-derived urinary furan metabolites Lys-BDA (l-2-amino-6-(2,5-dihydro-2-oxo-1H-pyrrol-1-yl)hexanoic acid), AcLys-BDA (l-2-(acetylamino)-6-(2,5-dihydro-2-oxo-1H-pyrrol-1-yl)hexanoic acid) and GSH-BDA (*N*-[4-carboxy-4-(3-mercapto-1H-pyrrol-1-yl)-1-oxobutyl]-l-cysteinyl-glycine cyclic sulfide), as well as acetyl acrolein (AcA, 2-oxo-pent-2-enal)-derived metabolites Lys-AcA (l-2-(acetylamino)-6-(2,5-dihydro-5-methyl-2-oxo-1H-pyrrol-1-yl)-hexanoic acid) and AcLys-AcA (l-2-amino-6-(2,5-dihydro-5-methyl-2-oxo-1H-pyrrol-1-yl)-hexanoic acid) and their stable isotopically labeled analogs, were synthesized and characterized through NMR and MS, and a stable isotope dilution analysis (SIDA) with UPLC-ESI-MS/MS was established. As a proof of concept, urinary samples of a four-day human intervention study were used. In the frame of this study, ten subjects ingested 500 mL of coffee containing 0.648 µmol furan and 1.059 µmol 2-MF. Among the furan metabolites, AcLys-BDA was the most abundant, followed by Lys-BDA and GSH-BDA. Exposure to 2-MF via the coffee brew led to the formation of Lys-AcA and AcLys-AcA. Within 24 h, 89.1% of the ingested amount of furan and 15.4% of the ingested amount of 2-MF were detected in the urine in the form of the investigated metabolites. Therefore, GSH-BDA, Lys-BDA, AcLys-BDA, Lys-AcA and AcLys-AcA may be suitable as short-term-exposure biomarkers of furan and 2-MF exposure.

## 1. Introduction

Furan and its alkylated analog 2-methylfuran (2-MF) are highly flammable aromatic-oxygen-containing heterocycles. In addition to their industrial use as basic chemicals, they are formed during combustion processes and thermal food processing. The formation of furan and 2-MF in food is complex because they may derive from compounds such as ascorbic acid, sugar, amino acids, polyunsaturated fatty acids and carotenoids [1,2,3] during food processing and cooking [4]. In particular, canned and baby food as well as coffee are high in furan and 2-MF [5]. In fact, for adults, coffee is the main source of dietary furan and 2-MF exposure, with 2-MF being the higher of the two [2]. The extent of formation of furan and 2-MF during coffee roasting depends on the roasting conditions [6]. Due to the volatility of furan, the use of a low temperature and long roasting time reduces the final furan and/or 2-MF content. In addition to process-dependent factors, storage conditions and household preparation methods of coffee beverages influence the content [7]. It has been estimated that only about 10% of the furan produced during roasting may be available for intake by coffee drinkers [6,8]. However, a medium-roast coffee brew may still contain, on average, 40 µg/kg of furan and 173 µg/kg of 2-MF [2,5]. Becalski et al. analyzed the mean concentrations of furan (38.7 µg/kg) and 2-MF (172 µg/kg) in 48 different non-brewed and brewed coffee samples [9]. They found a loss of furans of about 27–85% in brewed samples compared to non-brewed samples. Also, the concentration of 2-MF exceeded the concentration of furan in roasted coffee and the respective brews by approximately fourfold [9,10]. For risk assessment, it is important to establish valid biomarkers that reflect actual exposure as well as monitor the furan and 2-MF content in food.

Furan has been classified by the International Agency for Research on Cancer (IARC) as possibly carcinogenic to humans (group 2B) [11]. Furan and 2-MF have been shown to exert dose-dependent liver toxicity and carcinogenic effects in rodent studies [2,12]. However, it is not yet clear whether furan or 2-MF exerts a genotoxic effect, as the results of in vivo and in vitro genotoxicity tests are inconclusive [2,13]. After oral intake, furan and 2-MF are absorbed from the gastrointestinal tract. It has been reported that when radiolabeled [2,5-^14^C]-furan (8 mg/kg bodyweight (bw)) was administered to rats via gavage, approximately 80% of the radioactivity was eliminated within 24 h, of which 14% was exhaled as furan and 26% as CO_2_, and 20% was detected in urine and 22% in feces [14]. After absorption, furan is metabolized by cytochrome P450 2E1 to *cis*-2-buten-1,4-dial (BDA) [15].

**Figure 1:**
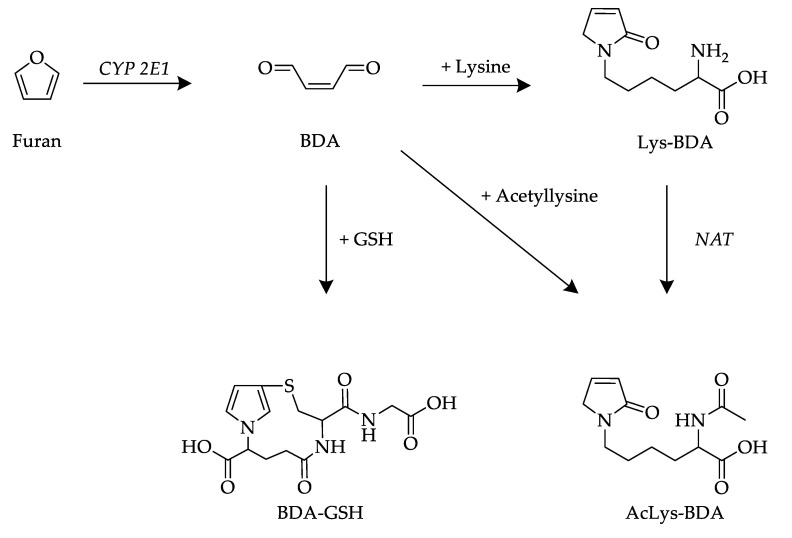
Metabolic pathway and formation of the investigated renal furan metabolites [17,21,22].

BDA is highly reactive and reacts with cellular nucleophiles, such as proteins, amino acids, nucleosides and glutathione (GSH), as shown in Figure 1 [1]. Metabolites detected in urine have been shown to be related to BDA. A renal metabolite in the urine of furan-treated rats was identified as GSH-BDA (*N*-[4-carboxy-4-(3-mercapto-1H-pyrrol-1-yl)-1-oxobutyl]-l-cysteinylglycine cyclic sulfide) [16]. GSH-BDA has also been detected in rat urine through metabolite profiling together with other metabolites, such as AcLys-BDA (l-2-(acetylamino)-6-(2,5-dihydro-2-oxo-1H-pyrrol-1-yl)hexanoic acid) [17]. GSH-BDA can further react with amino acids such as Lys to form crosslinks. The degradation of these crosslinks can generate the metabolite AcCys-BDA-AcLys and its sulfoxide [18,19]. In rat urine, it was found that 0.6–1.1% of the applied furan dose was excreted renally as GSH-BDA and 1.4–2.1% as AcLys-BDA within 24 h [20].

However, no quantitative data have been reported on excretion in humans after dietary furan exposure. After furan inhalation through tobacco smoking, it was found that human acetylate Cys-BDA-Lys crosslinks mostly at the cysteine site. The corresponding sulfoxide (AcCys-BDA-Lys sulfoxide) showed a strong correlation with smoking and its level decreased after smoking cessation, revealing delayed kinetics and a potential contribution from the degradation of protein adducts [23]. These findings were confirmed through an analysis of urine samples from water pipe tobacco smokers [24].

In another study, when ^14^C-radiolabeled 2-MF (50–200 mg/kg bw) was administered to rats intraperitoneally (i.p.), radioactivity was detected in the liver, kidneys, lungs and blood [25]. After absorption, 2-MF is metabolized to 3-acetyl acrolein (AcA, 2-oxopent-2-enal) in rodents [2,25]. AcA is highly reactive and can be trapped with semicarbazide, but as a Michael acceptor, it can react with cellular nucleophiles, such as proteins, amino acids, nucleosides and glutathione (GSH).

**Figure 2:**
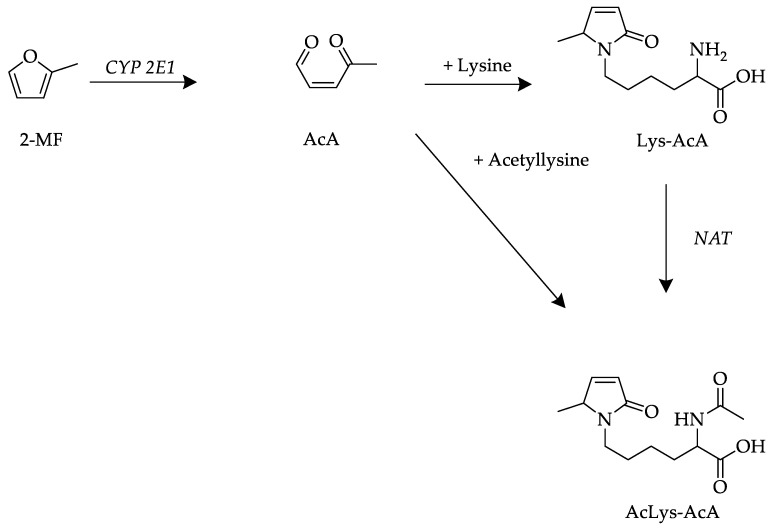
Proposed metabolic pathway and formation of the investigated renal 2-methylfuran (2-MF) metabolites (AcA formation first described by Ravindranath et al. [26]).

A proposed metabolic route from 2-MF to Lys-AcA and AcLys-AcA is shown in Figure 2.

To date, only limited nonquantitative data have been obtained through high-resolution ToF-MS on excretion in humans after dietary 2-MF exposure [27]. A correlation between smoking and methylfuran excretion was found by analyzing the volatile compounds furan, 2-MF and 3-methylfuran in urine samples [28].

The main aims of our study were to develop a quantitative method for the analysis of furan and 2-MF biomarkers in human urine and to provide first insights into furan and 2-MF metabolism after coffee intake.

## 2. Materials and Methods

### 2.1. Materials

For use in syntheses, 2,5-dihydro-2,5-dimethoxyfuran (97%, mixture of *cis* and *trans*, DMDHF), 2,5-dihydro-2,5-dimethoxy-2-methylfuran (97%, mixture of *cis* and *trans*, MDMDHF), l-Lys (≥98%), sodium hydroxide, sodium bicarbonate, acetic acid anhydride, GSH (≥98%), [Gly-^13^C_2_,^15^N]-GSH trifluoroacetate (^15^N ≥ 98%, ^13^C ≥ 99%, CP ≥ 95%) and *N*-α-acetyllysine were purchased from Sigma-Aldrich (St. Louis, USA), whereas acetic acid (99%) was from Avantor Performance Materials B.V. (Radnor, USA) and [^13^C_6_,^15^N_2_]-Lys x 2HCl (^13^C_6_ ≥ 99%; ^15^N_2_ ≥ 99%, CP ≥ 98%) was from Carl Roth (Karlsruhe, Germany). For preparative purposes, HPLC-grade formic acid and acetonitrile were purchased from Sigma-Aldrich (St. Louis, MO, USA) and Fisher (Schwerte, Germany), respectively, whereas MS-grade solvents formic acid and acetonitrile were purchased from Carl Roth (Karlsruhe, Germany) and Merck (Darmstadt, Germany), respectively. Ammonium formate was obtained from Fisher (Schwerte, Germany); ammonium acetate, D_2_O, DMSO-d_6_, sodium acetate and ammonia were from Sigma-Aldrich (St. Louis, MO, USA); ammonium hydroxide was from Grüssing (Filsum, Germany); and citric acid was from Carl Roth (Karlsruhe, Germany).

### 2.2. Syntheses

#### 2.2.1. Synthesis of l-2-Amino-6-(2,5-dihydro-2-oxo-1H-pyrrol-1-yl)hexanoic acid (Lys-BDA) and [^13^C_6_,^15^N_2_]-l-2-Amino-6-(2,5-dihydro-2-oxo-1H-pyrrol-1-yl)hexanoic acid ([^13^C_6_,^15^N_2_]-Lys-BDA)

An amount of 33.2 µL (273 µmol) of DMDHF was dissolved in 3 mL of 0.1% aqueous acetic acid and stirred for 24 h at room temperature (RT). This initial BDA formation method was based on Alves et al. [29], but acetic acid was used instead of HCl. An equimolar amount of l-Lys (40 mg, 273 µmol) or [^13^C_6_,^15^N_2_]-Lys (50 mg, 273 µmol) was dissolved in 1 mL of H_2_O_dd_, added to the BDA reaction solution and then stirred for another 24 h at RT. In the case of [^13^C_6_,^15^N_2_]-Lys, the hydrochloride was neutralized with NaOH before adding to the BDA reaction solution. Purification of the desired Lys-BDA adduct was performed in two runs through semi-preparative HPLC using a 1200 series Agilent HPLC system (Waldbronn, Germany) equipped with a Synergi 4 µm Polar-RP column (250 × 10 mm, Phenomenex, Torrance, CA, USA). In the first run, the synthesis mixture was diluted 1:5 with H_2_O_dd_ and injected in aliquots of 2 mL, and a mobile phase of 0.1% aqueous formic acid (A) and acetonitrile (B) was used at a flow rate of 5 mL/min. The gradient started at 1% B and was kept isocratic for 1.5 min, and then B was increased to 30% over 13 min. Afterward, the HPLC column was rinsed with 50% B for 2.8 min to prepare for the next cycle, which was started after at least 2.8 min of equilibration under the initial solvent conditions. Fractions of 0.25 min were collected and analyzed through HPLC-ESI-MS/MS (HPLC 1100 series, Agilent, coupled to a Sciex MS API 2000 (AB SCIEX, Darmstadt, Germany)). The desired product 2-amino-6-(2-oxo-5H-pyrrol-1-yl)-hexanoic acid (Lys-BDA) eluted in the time span of 6.25–9.00 min. Pooled fractions were purified again through the HPLC method described above, but H_2_O_dd_ was used as solvent A. The product eluted in a time interval of 6.00–8.25 min. After lyophilization, the yield was 21%. Characterization of Lys-BDA was performed through MS and NMR. [^13^C_6_,^15^N_2_]-Lys-BDA was only examined through MS. Purity was >95%.

l-2-Amino-6-(2,5-dihydro-2-oxo-1H-pyrrol-1-yl)hexanoic acid (*M* = 212.245 g/mol): ^1^H-NMR (400 MHz, D_2_O) *δ* [ppm]: 7.26 (dt, 1H, C3-H), 6.08 (dt, 1H, C4-H), 4.12 (bs, 2H, C5-H2), 3.64 (t, 1H, Lys α-CH), 3.41 (t, 2H, Lys ε-CH_2_), 1.90–1.72 (m, 2H, Lys β-CH_2_), 1.60 (quin, 2H, Lys δ-CH_2_), 1.39–1.20 (m, 2H, Lys γ-CH_2_); HPLC-ESI^pos^-MS^2^ of *m*/*z* 213 (CE = 30 eV): 82, 84, 132, 149, 167; *t*_R_ at 9.7 min.

[^13^C_6_,^15^N_2_]-l-2-amino-6-(2,5-dihydro-2-oxo-1H-pyrrol-1-yl)hexanoic acid (*M* = 220.188 g/mol): HPLC-ESI^pos^-MS^2^ of *m*/*z* 221 (CE = 30 eV): 88, 90, 138, 156, 174.

#### 2.2.2. Synthesis of l-2-(acetylamino)-6-(2,5-dihydro-2-oxo-1H-pyrrol-1-yl)hexanoic acid (Nα-AcLys-BDA) and [^13^C_6_,^15^N_2_]-l-2-(acetylamino)-6-(2,5-dihydro-2-oxo-1H-pyrrol-1-yl)hexanoic acid ([^13^C_6_,^15^N_2_]-Nα-AcLys-BDA)

Isolated Lys-BDA or [^13^C_6_,^15^N_2_]-Lys-BDA was dissolved in 0.5 mL of H_2_O_dd_ and mixed with 0.5 mL of a saturated sodium bicarbonate solution. Next, a 100-fold excess of acetic anhydride was added and then the mixture was stirred for 5 min at RT. Isolation was performed using the two-step HPLC method described above. In the first run, the desired product 2-acetamido-6-(2-oxo-5H-pyrrol-1-yl)hexanoic acid (Nα-AcLys-BDA) eluted between 11.5 and 13.0 min and in the second run between 7.0 and 12.0 min. After drying, a white powder was obtained with a yield of 64%. Purity (^1^H-NMR) was 96%.

l-2-(acetylamino)-6-(2,5-dihydro-2-oxo-1H-pyrrol-1-yl)hexanoic acid (*M* = 254.282 g/mol): ^1^H-NMR (400 MHz, D_2_O) *δ* [ppm]: 7.23 (dt, 1H, C3-H), 6.05 (dt, 1H, C4-H), 4.17 (dd, 1H, Lys α-CH), 4.07 (s, 2H, C5-H_2_), 3.37 (t, 2H, Lys ε-CH_2_), 1.91 (s, 3H, COCH_3_), 1.8–1.71 (m, 1H, Lys β-CH_a_), 1.69–1.59 (m, 1H, Lys β-CH_b_), 1.57–1.51 (m, 2H, Lys δ-CH_2_), 1.29–1.19 (m, 2H, Lys γ-CH_2_); HPLC-ESI^pos^-MS^2^ of *m*/*z* 255 (CE = 30 eV): 82, 84, 167, 209, 237; *t*_R_ at 13.2 min.

[^13^C_6_,^15^N_2_]-l-2-(acetylamino)-6-(2,5-dihydro-2-oxo-1H-pyrrol-1-yl)hexanoic acid: (*M* = 262.224 g/mol); HPLC-ESI^pos^-MS^2^ of *m*/*z* 263 (CE = 30 eV): 88, 90, 174, 216, 245.

#### 2.2.3. Synthesis of N-[4-carboxy-4-(2-or3-mercapto-1H-pyrrol-1-yl)-1-oxobutyl]-l-cysteinylglycine cyclic sulfide (GSH-BDA) and [Gly-^13^C_2_,^15^N]-N-[4-carboxy-4-(2-or3-mercapto-1H-pyrrol-1-yl)-1-oxobutyl]-l-cysteinylglycine cyclic sulfide ([Gly-^13^C_2_,^15^N]-GSH-BDA)

As described by Alves et al. [29], to the BDA solution prepared as described above, 1 mL of H_2_O_dd_ was added in which an equimolar amount of GSH or [Gly-^13^C_2_,^15^N]-GSH was dissolved. The reaction mixture was stirred for 24 h at RT. After 1:2 dilution with H_2_O_dd_, 2 mL of the solution was injected into the preparative HPLC system described above. Using a flow rate of 5 mL/min, the gradient started at 10% B, was kept isocratic for 1 min and was then raised to 40% B over 8 min. Afterward, the column was rinsed with 90% B for 5 min and equilibrated for an additional 5 min under the initial conditions. In the first cycle, 0.1% aqueous formic acid was used as solvent A, whereas in the second cycle, it was changed to H_2_O_dd_. The desired compounds *N*-[4-carboxy-4-(3-mercapto-1H-pyrrol-1-yl)-1-oxobutyl]-l-cysteinylglycine cyclic sulfide and *N*-[4-carboxy-4-(2-mercapto-1H-pyrrol-1-yl)-1-oxobutyl]-l-cysteinylglycine cyclic sulfide (BDA-GSH) eluted between 5.0 and 5.5 min. After freeze-drying, fractions containing a 1:1 mixture of both isomers were obtained with a yield of 2%. Purity was >95%.

BDA-GSH (*M* = 355.366 g/mol): ^1^H NMR (400 MHz, D_2_O) *δ* [ppm]: 6.89 (s, 1 H, pyrrole C5-Hα), 6.76 (s, 1 H, pyrrole C5-Hb), 6.71 (s, 1 H, pyrrole C2-H), 6.60 (s, 1 H, pyrrole C3-H), 6.27 (s, 1 H, pyrrole C4-Hb), 6.10 (s, 1 H, pyrrole C4-Hα), 3.89–3.75 (m, 4 H, Gly CH), 3.35–3.25 (m, 1 H, Cys β-CH_2A_), 2.9 (d, 1 H, Cys β-CH_2B_), 2.86–2.70 (m, 2 H, Cys β-CH_1_), 2.50–1.85 (m, 8 H, Glu β-CH_2_ + Glu γ-CH_2_); HPLC-ESI_neg_-MS^2^ of *m*/*z* 354 (CE = −31 eV): 98, 124, 141, 185. *t*_R_ at 12.7 min.

[^13^C_2_,^15^N]-BDA-GSH (*M* = 358.344 g/mol): HPLC-ESI^neg^-MS^2^ of *m*/*z* 357 (CE = −31 eV): 98, 124, 143, 188.

#### 2.2.4. Synthesis of l-2-Amino-6-(2,5-dihydro-5-methyl-2-oxo-1H-pyrrol-1-yl)hexanoic acid (Lys-AcA) and [^13^C_6_,^15^N_2_]-l-2-Amino-6-(2,5-dihydro-5-methyl-2-oxo-1H-pyrrol-1-yl)hexanoic acid ([^13^C_6_,^15^N_2_]-Lys-AcA)

An amount of 39.5 µL (273 µmol) of MDMDHF was dissolved in 3 mL of 0.1% aqueous acetic acid and stirred for 1 h at RT to obtain a 3-acetyl acrolein (AcA) reaction solution, similarly to the formation of the BDA reaction solution. An equimolar amount of l-Lys (40 mg, 273 µmol) or [^13^C_6_,^15^N_2_]-Lys (50 mg, 273 µmol) was dissolved in 1 mL of H_2_O_dd_, added to the AcA reaction solution and then stirred for another 24 h at RT. In the case of [^13^C_6_,^15^N_2_]-Lys, the hydrochloride was neutralized with NaOH before adding to the AcA reaction solution. Purification of the desired Lys-AcA adduct was performed in two runs through semi-preparative HPLC using an Agilent 1200 series HPLC system (Waldbronn, Germany) equipped with a Synergi 4 µm Polar-RP column (250 × 10 mm, Phenomenex, Torrance, USA). In the first run, the synthesis mixture was diluted 1:2 with H_2_O_dd_ and injected in aliquots of 2 mL using a mobile phase of 0.1% aqueous formic acid (A) and acetonitrile (B) at a flow rate of 6 mL/min. The gradient started at 1% B and was kept isocratic for 2.5 min, and then the percentage of B was increased to 8% over 7 min. Afterward, the HPLC column was rinsed with 80% B for 4.0 min to prepare for the next cycle, which was started after at least 5.0 min of equilibration under the initial solvent conditions. Fractions of 0.5 min were collected and analyzed through HPLC-ESI-MS/MS (HPLC 1100 series, Agilent, coupled to a Sciex MS API 2000 (AB SCIEX, Darmstadt, Germany)). The desired product l-2-amino-6-(2,5-dihydro-5-methyl-2-oxo-1H-pyrrol-1-yl)-hexanoic acid (Lys-AcA) eluted in a time span of 4.5–5.5 min. Pooled fractions were purified again via the HPLC method described above, but H_2_O_dd_ was used as solvent A. The product eluted in a time interval of 4.5–8.0 min. After lyophilization, the yield was 18%. Characterization of Lys-AcA was performed through MS and NMR. [^13^C_6_,^15^N_2_]-Lys-AcA was only examined through MS. Purity was > 95%.

l-2-Amino-6-(2,5-dihydro-5-methyl-2-oxo-1H-pyrrol-1-yl)hexanoic acid (*M* = 226.272 g/mol): ^1^H NMR (600 MHz, D_2_O) *δ* [ppm]: 3.62 (m, 1H, C5-H), 6.08 (dt, 1H, C4-H), 4.12 (bs, 2H, C5-H_2_), 3.15 (m, 1H, Lys α-CH), 2.45–2.33 (m, 2H, Lys ε-CH_2_), 2.17–2.00 (m, 2H, Lys β-CH_2_), 1.78–1.50 (m, 2H, Lys δ-CH_2_), 1.53–1.31 (m, 2H, Lys γ-CH2), 1.43 (d, 3H, CH_3_); HPLC-ESI_pos_-MS^2^ of *m*/*z* 227 (CE = 30 eV): 82 (69%), 84 (100%), 130 (19%), 163 (14%), 181 (25%). *t*_R_ at 8.8 min.

[^13^C_6_,^15^N_2_]-l-2-amino-6-(2,5-dihydro-5-methyl-2-oxo-1H-pyrrol-1-yl)hexanoic acid (*M* = 233.215 g/mol): HPLC-ESI_pos_-MS^2^ of *m*/*z* 235 (CE = 25 eV): 88, 90, 130, 170, 188.

#### 2.2.5. Synthesis of l-2-(acetylamino)-6-(2,5-dihydro-2-oxo-5-methyl-1H-pyrrol-1-yl)hexanoic acid (AcLys-AcA) and [^13^C_6_,^15^N_2_]-l-2-(acetylamino)-6-(2,5-dihydro-2-oxo-5-methyl-1H-pyrrol-1-yl)hexanoic acid ([^13^C_6_,^15^N_2_]-AcLys-AcA)

As described in Alves et al. [29] but with modifications, the isolated Lys-AcA or [^13^C_6_,^15^N_2_]-Lys-AcA was dissolved in 1 mL of H_2_O_dd_ and mixed with 1 mL of a saturated sodium bicarbonate solution. Next, a 100-fold excess of acetic anhydride was added, and the mixture was stirred for 5 min at RT. Isolation was performed using the two-step HPLC method described above. Purification of the desired Lys-AcA adduct was performed in two runs through semi-preparative HPLC using an Agilent 1200 series HPLC system (Waldbronn, Germany) equipped with a Synergi 4 µm Polar-RP column (250 × 10 mm, Phenomenex, Torrance, CA, USA). For the first run, the synthesis mixture was diluted 1:2 with H_2_O_dd_ and injected in aliquots of 2 mL using a mobile phase of 0.1% aqueous formic acid (A) and acetonitrile (B) at a flow rate of 6 mL/min. The gradient started at 1% B and was kept isocratic for 1.5 min, and the percentage of B was increased to 11% over 7 min and then to 20% B over 3 min. Afterward, the HPLC column was rinsed with 80% B for 2.5 min to prepare for the next cycle, which was started after at least 5.0 min of equilibration under the initial solvent conditions. Fractions of 0.5 min were collected and analyzed through HPLC-ESI-MS/MS (HPLC 1100 series, Agilent, coupled to a Sciex MS API 2000 (AB SCIEX, Darmstadt, Germany)). Pooled fractions were purified again via the HPLC method described above, but H_2_O_dd_ was used as solvent A. The product eluted between 9.0 and 12.0 min. In the first run, the desired product l-2-acetylamino-6-(2,5-dihydro-2-oxo-5-methyl-5H-pyrrol-1-yl)hexanoic acid (AcLys-AcA) eluted between 9.5 and 11.5 min and, in the second run, between 8.0 and 12.0 min. After drying, a white powder was obtained with a yield of 68%. Purity (^1^H-NMR) of > 98%.

l-2-(acetylamino)-6-(2,5-dihydro-2-oxo-5-methyl-1H-pyrrol-1-yl)hexanoic acid (*M* = 268.308 g/mol): ^1^H-NMR (600 MHz, D_2_O): *δ*[ppm] 4.29 (dd, *J* = 8.7, 4.8, 1H) 3.26–3.17 (m, 2H), 3.15 (t, *J* = 6.7 Hz, 0H), 2.83 (t, *J* = 6.6 Hz, 0H), 2.56–2.48 (m, 1H), 2.45–2.36 (m, 1H), 2.26–2.20 (m, 1H), 2.19 (s, 0H), 2.12–2.04 (m, 1H), 2.01 (s, 3H), 1.91–1.81 (m, 1H), 1.78–1.69 (m, 1H), 1.63–1.54 (m, 2H), 1.50 (d, *J* = 10.0 Hz, 2H), 1.44–1.31 (m, 2H); HPLC-ESI_pos_-MS^2^ of *m*/*z* 269 (CE = 25 eV): HPLC-ESI_pos_-MS^2^ of *m*/*z* 269 (CE = 25 eV): 82 (26%), 84 (23%), 164 (100%), 181 (31%), 223 (11%), 227 (12%); *t*_R_ at 12.0 min.

[^13^C_6_,^15^N_2_]-l-2-(acetylamino)-6-(2,5-dihydro-5-methyl-2-oxo-1H-pyrrol-1-yl)hexanoic acid: (*M* = 276.251 g/mol); HPLC-ESI_pos_-MS^2^ of *m*/*z* 277 (CE = 25 eV): 88, 90, 171, 188, 230, 234.

### 2.3. Stability Study

To explore the influence of pH on the storage stability of the metabolites detected in the urine samples, a fresh urine sample was collected from a volunteer 3 to 4 h after they had consumed 500 mL of freshly brewed coffee. The urine sample was divided into 4 mL aliquots, and each was mixed with either 4 mL of 100 mM ammonium formate buffer at pH 10 or 4 mL of 100 mM ammonium acetate buffer at pH 5.4. The adjusted aliquots were stored at −20 °C and analyzed on the same day as collection and after 4, 8, 12, 24, 36, 48 and 60 weeks.

### 2.4. Human Intervention Study

This study was originally conducted to investigate the excretion of niacin and alkylpyrazine metabolites in 2015 and has been described in detail elsewhere [30,31]. However, due to the appropriate study design, it was also used as a pilot study for the investigation of renal excretion of furan and 2-MF metabolites after coffee intake. The human intervention study was approved by the ethical commission of the Rhineland-Palatinate medical commission in Mainz (no. 837.427.15(10195)) and performed in accordance with the ethical standards of the Declaration of Helsinki (1964).

Ten healthy, nonsmoking volunteers of Caucasian origin (six females and four males; age 22–27 years, BMI 22.6 ± 2.6 kg m^−2^) who were accustomed to drinking coffee on a daily basis participated in this study and gave written consent before joining this study. Other inclusion criteria were as follows: age between 20 and 50 years, BMI between 19 and 25 kg/m^2^, not taking medication and/or dietary supplements, not pregnant, not performing competitive sports, not participating in other studies or a regular blood donor. This study lasted four days, during which the participants received standardized meals prepared without canned foods and heated exclusively in open pots to minimize heat induced contaminant intake. For each participant, a first overnight urine sample was collected during the last 12 h of a two-day run-in period, followed by a spot urine sample in the morning prior to coffee consumption. Subsequently, each participant consumed 500 mL of freshly brewed coffee prepared from 30 g of medium–dark-roasted Arabica coffee (provided by Tchibo GmbH, Hamburg, Germany) using a SENSEO pad coffee maker (Philips, Amsterdam, Netherlands). An aliquot of the coffee brew was stored at −20 °C and then analyzed using GC-MS to determine its furan (44.11 µg, 0.648 µmol per 500 mL coffee) and 2-MF (86.94 µg, 1.059 µmol per 500 mL coffee) content. After coffee consumption, total urine was collected over a 36 h period divided into ten collection intervals: 0–1 h, 1–2 h, 2–3 h, 3–4 h, 4–5 h, 5–6 h, 6–8 h, 8–12 h, 12–24 h and 24–36 h. Volumes of urine were calculated by weighing the samples and using an assumed density of 1 kg/L. Aliquots were stored at −20 °C until sample preparation.

### 2.5. Sample Preparation

An internal standard working solution was prepared in H_2_O_dd_ containing 1.0 µM [^13^C_6_,^15^N_2_]-Lys-BDA, 2.5 µM [^13^C_6_,^15^N_2_]-AcLys-BDA, 1.0 µM [Gly-^13^C_2_,^15^N]-GSH-BDA, 2.5 µM [^13^C_6_,^15^N_2_]-Lys-AcA and 1.0 µM [^13^C_6_,^15^N_2_]-AcLys-AcA and was stored at −20 °C. Urine samples from the human intervention study were thawed at RT. An amount of 1 mL of each sample was spiked with 20 µL of the internal standard working solution. After acidification with 50 µL of formic acid, the samples were mixed well and centrifuged (15.000× *g*, 5 min). Afterward, the supernatants were transferred into brown glass vessels through a syringe filter (0.22 µm). Aliquots of 2 µL were analyzed through the HPLC-ESI-MS/MS method described below.

### 2.6. UPLC-ESI-MS/MS

An Agilent 1290 series HPLC system (Waldbronn, Germany) was used coupled to a QTRAP 5500 mass spectrometer (AB SCIEX, Darmstadt, Germany) equipped with an electrospray (ESI) source. The metabolites Lys-BDA, AcLys-BDA, GSH-BDA, Lys-AcA and AcLys-AcA and their stable-isotope-labeled analogs used as standards were separated using a Synergi 2.5 µm Polar-RP column (100 × 2 mm, Phenomenex, Torrance, CA, USA) equipped with an appropriate guard column at 50 °C. The mobile phase comprised 0.1% aqueous formic acid (A) and 0.1% formic acid in acetonitrile (B) at a flow rate of 400 μL/min. The content of B in the eluent was initially held for 0.2 min at 1% and then was raised to 7% over 0.8 min and held for 1.9 min. Afterward, the content of B was increased to 35% over 0.3 min. The final conditions were held for 0.7 min and then the column was rinsed with 95% B for 1.4 min and equilibrated for 2.5 min with 1% B. Due to different ionization conditions, two different MRM periods in the positive mode were used. Table 1 shows the MS parameters for all analyzed compounds, whereas Table 2 shows LOD and LOQ, which were defined as 1:3 and 1:10 of the signal-to-noise ratio, respectively [32].

On this basis, standard solutions for calibration were prepared in H_2_O_dd_ with concentration ranges of 5–250 nM for Lys-BDA, 10–500 nM for AcLys-BDA, 1–50 nM for GSH-BDA, 5–250 nM for Lys-AcA and 2–100 nM for AcLys-ACA containing 20 nM [^13^C_6_,^15^N_2_]-Lys-BDA, 50 nM [^13^C_6_,^15^N_2_]-AcLys-BDA, 20 nM [Gly-^13^C_2_,^15^N]-GSH-BDA, 50 nM [^13^C_6_,^15^N_2_]-Lys-AcA and 20 nM [^13^C_6_,^15^N_2_]-AcLys-AcA. For calibration, the observed peak area ratios were plotted against the concentration ratios, resulting in a correlation coefficient of *R*^2^ > 0.99. The method’s precision was determined by repeatedly analyzing standards at LOQ, at the highest concentration used for calibration and at the midway point between these two values (Table 2). The intra-day coefficients of variation were ≤4.1% for the intermediate and high concentrations, and ≤5.7% for LOQ. The corresponding inter-day coefficients of variation were ≤5.1% based on analyses performed on five consecutive days. Using a spiked urine sample, the robustness was shown to be below 8.4% for all analytes under study. Recoveries were between 99 and 103%.

### 2.7. Data Analysis/Statistics

Each urine sample was analyzed in triplicate. The HPLC-ESI-MS/MS system was operated using the Analyst 1.7.2 software package (AB Sciex, Darmstadt, Germany), and chromatographic data were processed using Multiquant 3.0.3 (AB Sciex, Darmstadt, Germany). Quantitative data for the urine samples are reported as the mean ± standard deviation (SD) for all ten participants. Two-sample *t*-tests were performed using Origin, Version 2023 (OriginLab Corporation, Northampton, MA, USA); statistical significance was determined using *p*-value thresholds of *p* ≤ 0.05, *p* ≤ 0.01 and *p* ≤ 0.001.

## 3. Results

In a previous untargeted approach [27], urine samples were analyzed and for the first time shown to contain metabolites of 2-MF, i.e., AcLys-AcA, Lys-AcA and AcCys-AcA, and additionally acetylcysteine butendial lysine (AcCys-BDA-Lys), acetylcysteine-SO-butendial acetyllysine (AcCys-(SO)-BDA-AcLys), methanethiol butendial glutamic acid (methanethiol-BDA-Glu), glutathione butendial lysine (GSH-BDA-Lys) and glutathione butendial glutamine (GSH-BDA-Gln). These metabolites were only identified through high-resolution MS but could not be quantified. In the present study, urine samples were analyzed, with the most abundant peaks identified as Lys-BDA, AcLys-BDA and GSH-BDA as metabolites of furan and AcLys-AcA, Lys-AcA and AcCys-AcA as metabolites of 2-MF using UPLC-ESI-MS/MS. The proposed formation mechanisms are shown in Figure 1 and Figure 2. Afterward, these analytes and their isotopically labeled analogs were synthesized, and a robust UPLC-ESI-MS/MS method was established. Other metabolites identified by Stegmüller et al. [27] using high resolution could not be clearly confirmed by the respective synthesis of standards, e.g., AcCys-BDA-Lys, and due to occurring mass defects, a clear identification was not possible. Urine samples collected during the human intervention study were assayed using the method described above and analytes were quantified through SIDA in the MRM mode (see experimental section). To accurately determine the amounts of furan metabolites Lys-BDA, AcLys-BDA and GSH-BDA as well as 2-MF metabolites Lys-AcA and AcLys-AcA in human urine samples after coffee intake, a stable isotope dilution analysis (SIDA) with HPLC-ESI-MS/MS detection was developed. Therefore, reference material for analytes that were not commercially available, as well as the corresponding stable-isotope-labeled analogs, was synthesized as internal standards for the SIDA.

### 3.1. Synthesis

The most pronounced 2-MF metabolites AcLys-AcA and Lys-AcA as well as the isotopically labeled analogs were synthesized in accordance with the method already established for the synthesis of AcLys-BDA, Lys-BDA and GSH-BDA published by Alves et al. [29] with modifications.

According to protocols in the literature, BDA can be synthesized from furan using dimethyldioxirane [33] and 2,5-diacetoxy-2,5-dihydrofuran (DMDHF) [34]. However, for the syntheses in this work, BDA was generated from DMDHF under acidic conditions [29]. The reaction of BDA with amino groups can yield both 3-pyrrolin-2-one and 4-pyrrolin-2-one derivatives. In preliminary experiments using 2,5-diacetoxy-2,5-dihydrofuran and Lys, these two isomers were formed at each of the two amino groups of Lys (data not shown). The elution order of the four isomers on the HPLC column (Synergi 4 µm Polar-RP, Phenomenex, Torrance, CA, USA) was 4-pyrrolin-2-one-α-Lys, 4-pyrrolin-2-one-ε-Lys, 3-pyrrolin-2-one-α-Lys and 3-pyrrolin-2-one-ε-Lys. In addition, other products whose mass spectra suggested BDA-Lys-BDA conjugates were observed during the synthesis. In contrast, the reaction of DMDHF produced only the desired 3-pyrrolin-2-one derivatives in a ratio of about 8:2 (ε-Lys/α-Lys). In the presence of strong acids and bases, a tautomeric equilibrium of the pyrrolin-2-one-derivatives was previously observed [35]. The opening of the pyrrolin-2-one ring structure under basic conditions has already been discussed by Alves et al. as a possible mechanism [36]. This is probably the reason why lyophilization under acidic conditions led to rearrangement of the desired compound and necessitated a solvent change after purification (first preparative HPLC run). A tautomeric rearrangement also occurs during acetylation. Hence, the isomers were purified again. The measured ^1^H-NMR spectrum of AcLys-BDA is in accordance with the literature data [37]. During the synthesis and purification of GSH-BDA, it was not possible to separate the two isomers (7-[(carboxymethyl)carbamoyl]-5-oxo-9-thia-1,6-diazabicyclo [8.2.1]trideca-10(13),11-diene-2-carboxylic acid and 3-[(carboxymethyl)carbamoyl]-5-oxo-3,4,5,6,7,8-hexahydro-2H-pyrrolo [2,1-b][1,3,8]thiadiazecine-8-carboxylic acid). According to NMR spectra, the isomers were present in a 1:1 mixture and the data are consistent with the literature [21]. Since the isomers could not be distinguished by either chromatography or mass spectrometry, it was not possible to clarify the ratio in which the two isomers were metabolically formed. The syntheses of the corresponding isotopically labeled compounds were analogous and confirmed through comparison of mass spectrometric data.

### 3.2. Urine Sample Analysis

**Figure 3:**
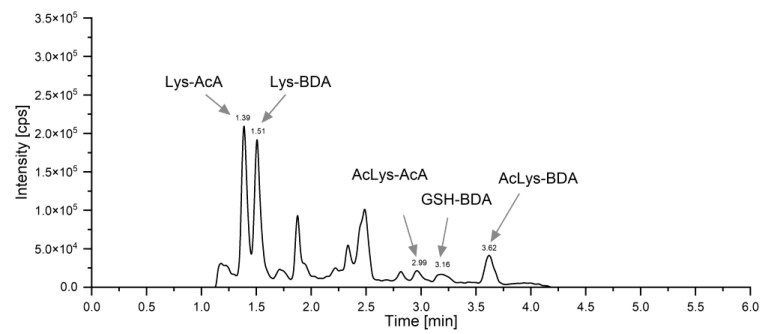
Total Ion Chromatogram (TIC) of a representative urine sample after ingestion of 500 mL of coffee brew containing 44.11 µg of furan and 86.94 µg of 2-MF (P3, time point 2–3 h). Respective retention times of Lys-BDA, AcLys-BDA and GSH-BDA (metabolites of furan), with AcLys-AcA and Lys-AcA (metabolites of 2-MF) indicated.

A representative chromatogram is shown in Figure 3. During sample preparation, only isotopically labeled standards were added, and the samples were vortexed, acidified and filtered prior to UPLC-ESI-MS/MS measurement to avoid spurious results.

**Figure 4:**
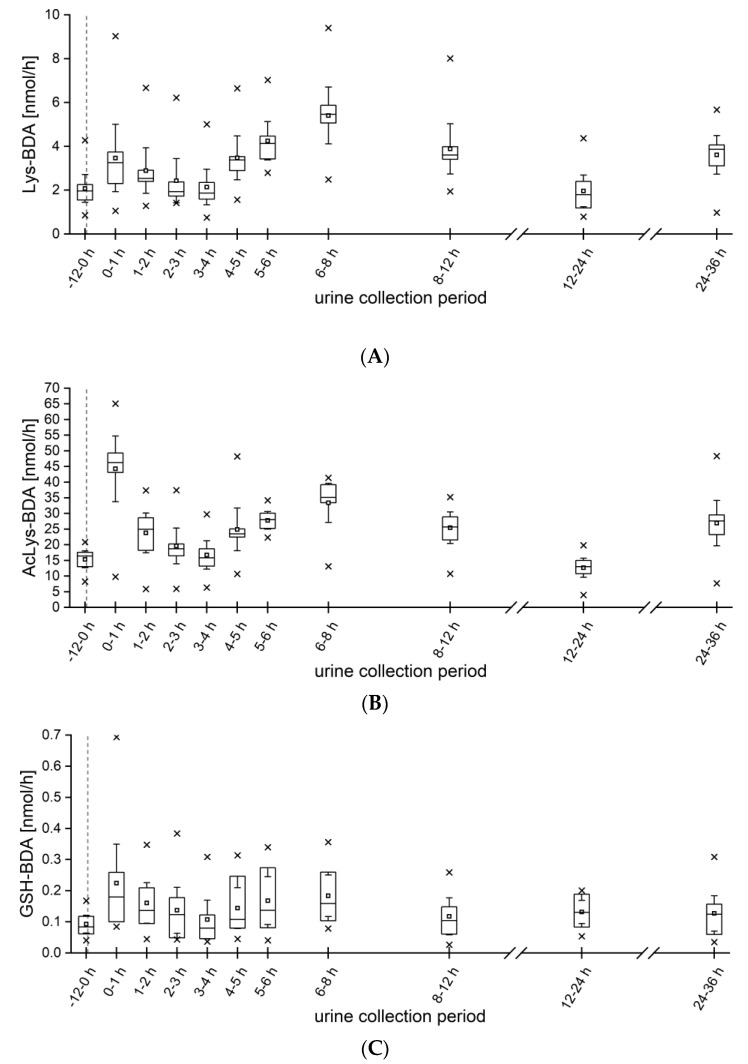
Excretion kinetics (nmol/h) of (**A**) Lys-BDA, (**B**) AcLys-BDA and (**C**) GSH-BDA from 12 h before to 36 h after ingestion of 500 mL of coffee brew. (*n* = 10) Boxes, Perc 25/75, whisker, 95% confidence interval; squares, mean; dash, median; times symbol, minimum and maximum; dashed gray line, coffee intake.

All three metabolites of furan (AcLys-BDA, Lys-BDA and GSH-BDA) were present in urine samples collected during the run-in period as well as after coffee consumption. The most abundant analyte was AcLys-BDA, followed by Lys-BDA and GSH-BDA. All three metabolites showed similar kinetics, as shown in Figure 4. For Lys-BDA, a slight but non-significant increase was detected in the first hour after coffee consumption. During the urine collection periods 4–5 h, 5–6 h, 6–7 h and 8–12 h, the excretion rate was significantly higher than the background value prior coffee ingestion, with a maximum at 6–8 h (5.4 ± 1.7 nmol/h). Within the first 24 h, 10.6 ± 4.4% of the ingested amount of furan was excreted as Lys-BDA. In contrast, the excretion rate of AcLys-BDA reached its maximum already after 1 h (44.3 ± 14.7 nmol/h), which was also highly significant compared to the background rate. A second maximum was detected in the period of 6–8 h (33.4 ± 8.7 nmol/h), which was comparable to the other two analytes. In total, the participants excreted 78 ± 18% (0.505 ± 0.117 µmol) of the ingested furan as AcLys-BDA in urine in the 24 h following intake. In furan-treated rats, the excretion of 1.4–2.1% of the dose was detected as AcLys-BDA within 24 h [20]. This seems low, but rats have been shown to only excrete 20% of the furan dose via urine [14]. The trend in kinetics of the metabolite GSH-BDA was very similar to that of Lys-BDA. There was a non-significant increase after 1 h (0.22 ± 0.18 nmol/h) and a significant maximum during the urine collection period of 6-8 h (0.18 ± 0.09 nmol/h). Overall, the participants excreted 89.1 ± 21.1% (0.577 ± 0.137 µmol) of the ingested furan in the urine within 24 h.

**Figure 5:**
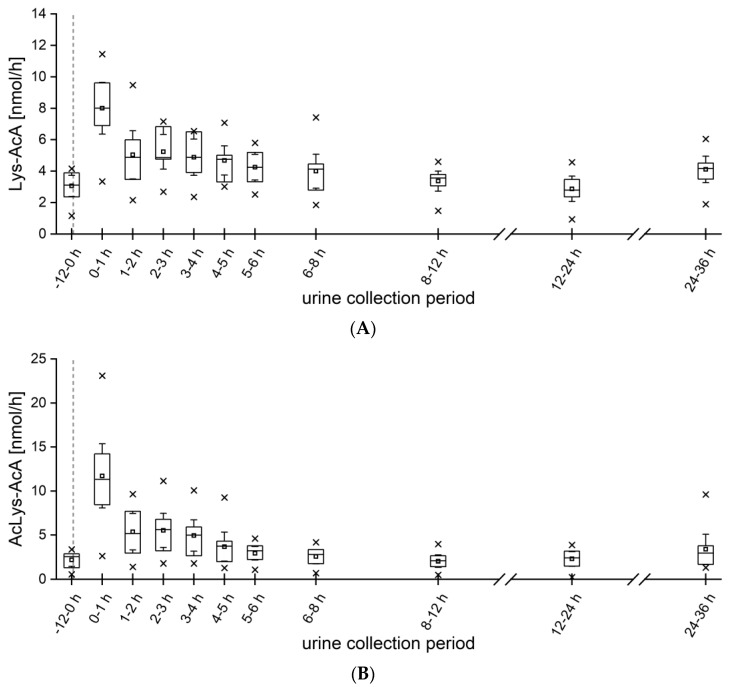
Excretion kinetics (nmol/h) of (**A**) Lys-AcA and (**B**) AcLys-AcA from 12 h before to 36 h after ingestion of 500 mL of coffee brew. (*n* = 10) Boxes, Perc 25/75, whisker, 95% confidence interval; squares, mean; dash, median; times symbol, minimum and maximum; dashed gray line, coffee intake.

For 2-MF, the two metabolites Lys-AcA and AcLys-AcA were monitored after coffee consumption, which both peaked already 1 h after ingestion. As shown in Figure 5, the metabolites showed similar kinetics. The maximum during 0–1 h for Lys-AcA was 8.0 ± 2.4 nmol and, for AcLys-AcA, 11.7 ± 5.4 nmol/h. Overall, 8.3 ± 2.4% (0.088 ± 0.025 µmol) of the ingested amount of 2-MF was excreted as Lys-AcA and 7.1 ± 2.8% (0.075 ± 0.030 µmol) as AcLys-AcA. In total, 15.4 ± 4.8% (0.163 ± 0.051 µmol) of the ingested 2-MF was excreted in the form of the metabolites under study. Interestingly, for the 2-MF metabolites, no second increase was observed in the kinetics, in contrast to the furan metabolites, which showed a slight second increase after 4 h. To the best of our knowledge, the kinetics of 2-MF metabolites in humans have not yet been reported. However, in 1985, Ravindranath et al. showed that AcA might be formed when scavenging agents such as N-acetyl cysteine (NAC), semicarbazide and cysteine are used [25]. Our study provides the first quantitative data on 2-MF metabolites, revealing similarities to the metabolism of furan.

**Figure 6:**
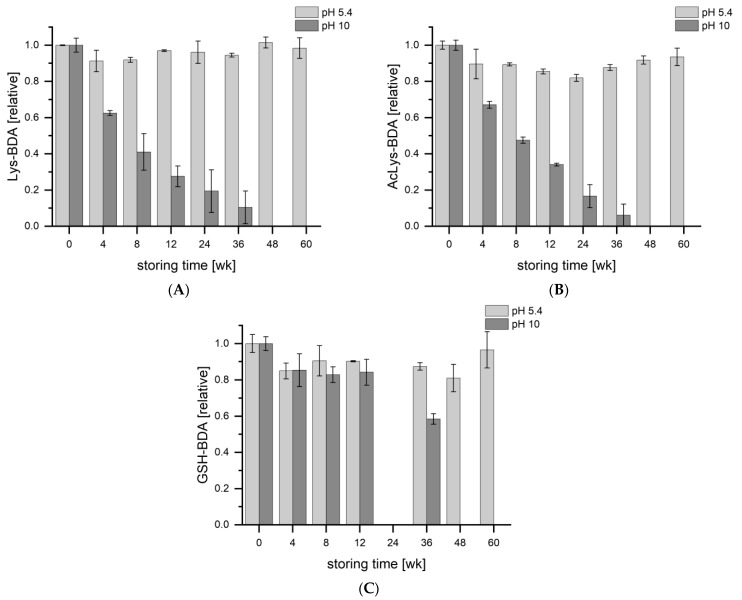
Stability of (**A**) Lys-BDA, (**B**) AcLys-BDA and (**C**) GSH-BDA stored at pH 5.4 and 10 over a period of 60 weeks at −20 °C. Data for GSH-BDA after 24 weeks are missing.

At the time of analysis, the samples were more than two years old and had been frozen unbuffered. The pH of the collected samples was not measured, but the pH of human urine typically ranges from 4.5 to 8.0 [38]. To verify whether pH-sensitive metabolites were degraded during storage and to optimize sample storage for further studies, the stability at different pH values was investigated over a period of 60 weeks (Figure 6). Storage at pH 10 resulted in significant losses of Lys-BDA and AcLys-BDA in the urine samples after only 4 weeks, and their levels continued to decrease thereafter. A possible mechanism for rearrangement of the pyrrolin-2-one ring structure has been discussed previously [36]. In contrast, storage at a slightly acidic pH of 5.4 did not result in significant reductions. Since the latter is a typical pH of natural human urine, it can be assumed that no significant losses occurred during the storage period. These results demonstrate the importance of controlling the pH during storage.

## 4. Discussion

Our pilot study had several limitations as this study was designed to determine niacin in urine after coffee consumption. The focus was on niacin and afterward on pyrazin metabolism in humans. As a first step, this study was used to identify markers of furan and alkylfuran metabolism. When interpreting metabolization rates, these facts have to be considered. For example, the subjects excreted furan and 2-MF metabolites above the baseline excretion even on day 4 without coffee consumption in the days before. At the end of the run-in phase, excretion of the metabolites was clearly detectable. This can probably be explained by the study design; although the subjects had a controlled diet that was low in heated foods to minimize levels of furan and 2-MF, they were not given any behavioral guidelines or restrictions. Thus, it is possible that exposure to furan and 2-MF may have occurred, e.g., through passive smoking, living and/or walking on a busy street, open fires or other unknown sources. This may also be the reason for interindividual variations. Grill et al. previously detected metabolites Lys-BDA and AcLys-BDA in the urine of nonsmokers [23]. Our results suggest that the furan metabolite AcLys-BDA and 2-MF metabolite AcLys-AcA may be suitable as biomarkers for short-term exposure to diet-associated levels of furan and 2-MF. Their significant increase in excretion immediately after the consumption of 500 mL of the coffee beverage may be indicative of the rapid detoxification of BDA and AcA via conjugation. In a recent study examining urine samples of waterpipe tobacco smokers in the morning before and after smoking, no significant differences were detected in the levels of six BDA metabolites, but levels of Lys-BDA and AcLys-BDA were higher after smoking than before [24]. This suggests that the latter metabolites are generated during smoking (Cys-BDA-Lys crosslinks and their derivatives), particularly as they were also found to be higher in the urine of smokers than of nonsmokers. Therefore, they may be suitable as biomarkers of long-term exposure because they could be released from proteins. In contrast, in our study, the increased excretion of AcLys-BDA immediately after coffee consumption suggests that it could be used as a biomarker for short-term exposure and originates from the reaction with free amino acids. However, further research is needed to clarify these findings [20]. Interestingly, in addition to the metabolites GSH-BDA and AcLys-BDA reported in rat urine samples after ingestion of furan [20], we identified and quantitated Lys-BDA.

## 5. Conclusions

This study provides the first insights into human metabolism and the urinary excretion of diet-related furan and 2-MF consumed in coffee. Despite this study’s limitations, AcLys-BDA, AcLys-AcA and Lys-AcA might have the potential to be viable biomarkers. However, there is an abundance of possible metabolites, as recently demonstrated in an untargeted analysis [27]. Therefore, further research is needed to identify and quantify additional metabolites of furan and 2-MF, e.g., dimethylfuran formed during food processing, and to perform human intervention studies considering, e.g., the influence of the food matrix on furan/2-MF bioavailability.

## Figures and Tables

**Table 1:** Mass spectrometric parameters for Lys-BDA, AcLys-BDA, GSH-BDA, Lys-AcA and AcLys-AcA, and their stable isotope (IS)-labeled analogs. Mass transitions labeled with * were used for quantification.

Compound-Specific Parameters		Rt (min)	Q1/Q3 (*m*/*z*)	Dwell (msec)	DP (V)	EP (V)	CE (V)	CXP (V)
**Period 1**	Lys-AcA	1.39	226.921/146.1 *	150	106	13	31	14
			226.921/181.2	75	106	13	17	18
	Lys-AcA-IS		234.887/90.0 *	150	86	10	27	10
			234.887/188.1	100	86	10	17	28
	Lys-BDA	1.51	212.847/84.1 *	125	31	8	29	8
			212.847/132.0	125	31	7	29	20
	Lys-BDA-IS		221.037/90.0 *	125	11	10	29	14
			221.037/174.1	125	11	10	17	20
**Period 2**	AcLys-AcA	2.98	268.859/164.0 *	90	131	13	25	16
			268.859/82.0	70	131	7	31	12
	AcLys-AcA-IS		276.944/170.1 *	90	136	11	33	14
			276.944/88.0	70	136	11	37	18
	GSH-BDA	3.16	355.953/210.0 *	175	36	10	31	16
			355.953/136.0	125	36	8	73	14
	GSH-BDA-IS		358.829/209.8 *	175	36	10	37	16
			358.829/136.0	125	36	10	71	8
	AcLys-BDA	3.62	254.962/84.0 *	90	56	12	39	10
			254.962/209.1	70	56	10	17	28
	AcLys-BDA-IS		262.946/90.1 *	90	41	10	35	10
			262.946/216.0	70	41	10	17	18
**MS-specific parameters**		**Duration** **(min)**	**CUR** **(psi)**	**IS** **(V)**	**TEM (°C)**	**GS1** **(psi)**	**GS2 (psi)**	**CAD**
**Period 1**		2.55	20	1500	550	45	55	−2
**Period 2**		3.45	20	5500	550	45	55	−3

IS, ionspray voltage; GS1, nebulizer gas; TEM, source temperature; GS2, heater gas; CUR, curtain gas; CAD, collisionally activated dissociation gas; DP, declustering potential; EP, entrance potential; CE, collision energy; CXP, cell exit potential.

**Table 2:** Parameters for validation of the method.

	LOD (nM)	LOQ (nM)	Standard conc. (nM)	Recovery (%)
Lys-BDA	0.14	0.47	1.2	102
AcLys-BDA	0.13	0.44	2.4	103
GSH-BDA	1.2	4.0	10	99
Lys-AcA	0.26	0.85	1.6	101
AcLys-AcA	0.21	0.70	2.0	102

## Data Availability

The data presented in this study are available on request from the corresponding author. Data available on request due to restrictions e.g., privacy or ethical.
